# Microbial Enzymes: Tools for Biotechnological Processes

**DOI:** 10.3390/biom4010117

**Published:** 2014-01-16

**Authors:** Jose L. Adrio, Arnold L. Demain

**Affiliations:** 1 Neol Biosolutions SA, BIC Granada, Granada 18016, Spain; E-Mail: jladrio@neolbio.com (J.L.A.); 2 Research Institute for Scientists Emeriti (R.I.S.E.), Drew University, Madison, NJ 07940, USA

**Keywords:** biocatalysis, industrial enzymes, microbial hosts, protein engineering

## Abstract

Microbial enzymes are of great importance in the development of industrial bioprocesses. Current applications are focused on many different markets including pulp and paper, leather, detergents and textiles, pharmaceuticals, chemical, food and beverages, biofuels, animal feed and personal care, among others. Today there is a need for new, improved or/and more versatile enzymes in order to develop more novel, sustainable and economically competitive production processes. Microbial diversity and modern molecular techniques, such as metagenomics and genomics, are being used to discover new microbial enzymes whose catalytic properties can be improved/modified by different strategies based on rational, semi-rational and random directed evolution. Most industrial enzymes are recombinant forms produced in bacteria and fungi.

## 1. Introduction

Microbial enzymes are known to play a crucial role as metabolic catalysts, leading to their use in various industries and applications. The end use market for industrial enzymes is extremely wide-spread with numerous industrial commercial applications [1]. Over 500 industrial products are being made using enzymes [2,3]. The demand for industrial enzymes is on a continuous rise driven by a growing need for sustainable solutions. Microbes have served and continue to serve as one of the largest and useful sources of many enzymes [4]. Many industrial processes, including chemical synthesis for production of chemicals and pharmaceuticals, have several disadvantages: low catalytic efficiency, lack of enantiomeric specificity for chiral synthesis, need for high temperature, low pH and high pressure. Also, the use of organic solvents leads to organic waste and pollutants. Enzymes are more useful for these applications as they work under mild reaction conditions (e.g., temperature, pH, atmospheric conditions), do not need protection of substrate functional groups, have a long half-life, a high stereo-selectivity yielding stereo- and regio-chemically-defined reaction products at an acceleration of 10^5^ to 10^8^-fold, and, in addition, they work on unnatural substrates [5]. Furthermore, enzymes can be selected genetically and chemically-modified to enhance their key properties: stability, substrate specificity and specific activity. There are drawbacks however, to the use of enzymes, e.g., certain enzymes require co-factors. However, various approaches such as cofactor recycling and use of whole cells can solve this problem. About 150 industrial processes use enzymes or whole microbial cell catalysts.

The global industrial enzymes market is very competitive with Novozymes being the largest player in the industry, followed by DSM, and DuPont (after it acquired a majority stake in Danisco and its Genencor division), among others. The companies mainly compete on the basis of product quality, performance, use of intellectual property rights, and the ability to innovate, among other such factors. North America and Europe are the largest consumers of industrial enzymes although the Asia Pacific region will undergo a rapid increase in enzyme demand in China, Japan and India, reflecting the size and strength of these country’s economies.

## 2. Discovering Enzymes

Nature provides a vast amount of microbial enzyme resources. Our ability to tap into such immense biodiversity depends on the tools available to expand the search for new enzymes by (i) metagenome screening [6,7,8,9,10]); (ii) genome mining in more than 2,000 sequenced microbial genomes [11,12,13,14]; and (iii) exploring the diversity of extremophiles [15,16]).

### 2.1. Metagenomic Screening

Although numerous microbes inhabit the biosphere, less than 1% can be cultivated through standard laboratory techniques. Metagenomics has appeared as an alternative strategy to conventional microbe screening by preparing a genomic library from environmental DNA and systematically screening such a library for the open reading frames potentially encoding putative novel enzymes [10,17].

Metagenomic screening is mostly based on either function or sequence approaches [8,10]. Function-based screening is a straightforward way to isolate genes that show the desired function by direct phenotypical detection, heterologous complementation, and induced gene expression [18]. On the other hand, sequence-based screening is performed using either the polymerase chain reaction (PCR) or hybridization procedures. Usually, the common procedure is to use a set of degenerated primers that have been designed based on consensus amino acid sequences.

Studies from different habitats such as volcanic vents, arctic tundra, cow rumen [19], marine environments [20], and termite guts [21] have yielded microbial enzymes with potential for biocatalytic applications, such as lipase [22,23], oxidoreductase [24], amidase [25], amylase [26], nitrilase [27], beta-glucosidase [28,29]), decarboxylase [30], and epoxide hydrolase [31].

Function-based screening of metagenomic libraries is somehow problematic mainly due to insufficient, biased expression of foreign genes in *Escherichia coli* as this bacterium is usually used as surogate host [8]. To overcome such hurdles, alternative bacterial host and expression systems are currently being examined including *Streptomyces lividans*, *Pseudomonas putida* and *Rhizobium leguminosarum*, among others [32,33].

Hit rate (probability of identifying a certain gene) also depends on other factors such as size of the target gene and the assay method. Enzyme activities are usually assayed on agar plates supplemented with different substrates Sensitivity of agar plate-based screening can be improved by using cell lysates [34], screening for genes giving resistance to toxic compounds [35,36], or linking the target activity to the expression of a reporter gene such as green fluorescent protein (GFP) [37] or β-galactosidase [38]. Also, development of flow cytometry based screens as SIGEX are leading the way as they enable more rapid screening of large metagenomic libraries [39]. Genes obtained through sequence-based screening are limited to those having homology to the sequences used as probes.

### 2.2. Microbial Genomes

Recent success of genome sequencing programs has resulted in an explosion of information available from sequence databases, thus creating an opportunity to explore the possibility of finding new natural products (including enzymes) by database mining [40]. The next generation sequencing platforms (454 from Roche, Solexa from Illumina or SoLiD from ABI), hold promise to reduce time and cost of genome sequencing. Using these platforms, as well as cutting edge approaches such as resequencing, helps to complete multiple whole genomes in less than two weeks. So far, more than 2,000 genome sequences and draft assemblies are available in the NCBI database [41].

Two approaches are being followed to discover new enzymes [42]. On one hand, genome hunting is based on searching for open reading frames in the genome of a certain microorganism. Sequences that are annotated as putative enzymes are subjected to subsequent cloning, over-expression and activity screening. Another approach, called data mining, is based on homology alignment among all sequences deposited in databases. Using different bioinformatics tools (e.g., BLAST), a search for conserved regions between sequences yields homologous protein sequences that are identified as possible candidates for further characterization.

### 2.3. Extremophiles

Over 30 articles on extremophiles have been published in a special issue [43]. Due to their capability to survive under environments of extreme conditions, both physical as temperature (−2 to 12 °C, 60–110 °C), pressure or radiation, and geochemical such as salinity (2–5 NaCl) and pH (<2, >9), extremophiles are a very interesting source of enzymes with extreme stability under conditions regarded as incompatible with biological materials. Recent studies show that the diversity of organisms in these extreme environments could be even greater than was initially thought [16,44]. However, because the majority of these microorganisms have not yet been isolated in pure culture, useful characterization of their enzymes still remains quite difficult.

Thermophilic proteases, lipases as well as cellulases and amylases are being used in many different industrial applications [16,45]. Extreme thermophiles (growing at 60–80 °C) are widely distributed among several bacterial genera such as *Clostridium*, *Thermus*, *Thermotoga*, and *Bacillus*, whereas most of hyperthermophiles belong to Archaea such as *Pyrococcus*, *Thermococcus* or *Methanopyrus*, among others. The Taq DNA polymerase, isolated from the thermophilic *Thermus aquaticus*, had 2009 sales of $500 million [46].

Also, enzymes from psychrophiles have become quite interesting for many industrial applications, partly because of the ongoing efforts to reduce energy consumption. Therefore, enzymes like proteases, amylases or lipases have great commercial potential for development of detergents to reduce wear and tear of textile fibers. Polymer-degrading activities (e.g., cellulases, xylanases,) are quite interesting for the pulp and paper industry, as well as for saccharification of pre-treated lignocellulosic biomass for the production of second generation biofuels [47]. These cold-active enzymes also have potential for many other applications [16,48], such as extraction and clarification of fruit juices, improvement of bakery products, polishing and stone-washing of textiles, and bioremediation of waters contaminated with oils or hydrocarbons [49].

Enzymes from halophites have to cope with very high concentrations of salts (e.g., sodium or potassium chloride). Such proteins have adapted to these environments by acquiring a large number of negatively-charged amino acid residues of their surfaces to prevent precipitation. Such a property has been taken advantage of by use of non-aqueous media [50]. Xylanases, amylases, proteases and lipases from *Halobacterium*, *Halobacillus* and *Halothermothrix* have been produced and their potential has been reviewed [51,52]).

Microorganisms that can survive under extreme pH values could be good sources of thermoalkaliphilic enzymes, like proteases and lipases, particularly useful for applications as additives in laundry and dishwashing detergents [16,53].

## 3. Strategies to Improve Properties of Microbial Enzymes

**Figure 1:**
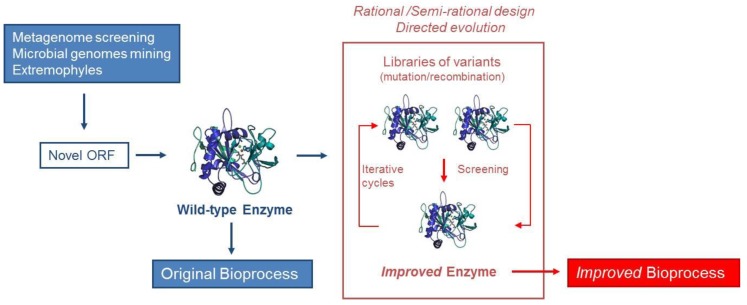
Discovery and development of biocatalysts.

The continuously expanding application of enzymes is creating a growing demand for biocatalysts that exhibit improved or new properties [46]. Although enzymes have favorable turnover numbers, they do not necessary fulfill all process requirements and need further fine tuning to achieve industrial scale production. Among those hurdles are: substrate/product inhibition, stability, narrow substrate specificity or enantioselectivity [54]. Genetic modification is very important and recombinant DNA techniques have increased production by 100-fold [55]. Development of new/improved biocatalysts is a challenging and complex task (Figure 1). There are two major ways in which enzymes can be modified to adapt their functions to applied ends: (i) rational redesign of existing biocatalysts and (ii) combinatorial methods which search for the desired functionality in libraries generated at random.

### 3.1. Rational Design

This approach includes site-directed mutagenesis to target amino acid substitutions, thus requiring knowledge of detailed information about the 3-dimensional structure and chemical mechanism of the enzymatic reaction, some of which may not be available. However, the increasing growth of databases containing protein structures and sequences is helping to overcome this lack of information. Comparison of the sequence of a new biocatalyst identified in a screening program with the thousands deposited in the databases can identify related proteins whose functions or/and structures are already known. Because new enzymes have evolved in nature by relatively minor modification of active-site structures, the goals of homology-driven experiments include engineering binding sites to fit different substrates as well as construction of new catalytic residues to modify functions and mechanisms [56]. A small number of variants are produced which are then screened. Although in many cases, results are poor compared to natural enzymes, there have been successes [57,58].

Computational protein design starts with the coordinates of a protein main chain and uses a force field to identify sequences and geometries of amino acids that are optimal for stabilizing the backbone geometry [59]. Because of the amazing number of possible sequences generated, the combination of predictive force fields and search algorithms is now being applied to functional protein design [60].

### 3.2. Directed Evolution

Combinatorial methods such as directed evolution create a large number of variants for screening for enantioselectivity, catalytic efficiency, catalytic rate, solubility, specificity and enzyme stability, but do not require extensive knowledge about the enzyme. Directed evolution is a fast and inexpensive way of finding variants of existing enzymes that work better than naturally occurring enzymes under specific conditions [3,61,62,63]. Directed evolution includes an entire range of molecular biological techniques that allow the achievement of genetic diversity mimicking mechanisms of evolution occurring in nature. It involves random mutagenesis of the protein-encoding gene by different techniques including the error-prone polymerase chain reaction (PCR) [64], repeated oligonucleotide directed mutagenesis [65], or chemical agents [66], among others. Error prone PCR accomplishes introduction of random point mutations in a population of enzymes. Such molecular breeding techniques (DNA shuffling, Molecular Breeding^TM^) allow *in vitro* random homologous recombination, typically between parent genes with homology higher than 70% [67]. After cloning and expression, a large collection of enzyme variants (10^4^–10^6^) is typically generated and is subjected to screening or selection.

All the approaches mentioned above are not mutually exclusive as the fields of rational, semi-rational and random redesign of enzymes are moving closer. Thus, directed evolution techniques make use, where possible, of smaller enzyme variant libraries designed by rational or semi-rational methods to reduce the screening effort but without compromising the likelihood of finding better variants [61,63]. One option is to target the active site residues (about 10–15 amino acids) and those closest to it (another 20–30 amino acids) as mutations closer to these regions seem to be more beneficial [68]. Another strategy, called CASTing, is based on a combinatorial active site testing [69], in which libraries are generated from groups of two or three residues made from the active site residues. Hits obtained from these initial libraries are combined and new libraries are generated and screened in an iterative way. By changing the nucleotide components during PCR, the amino acid alphabet can be reduced yielding new proteins composed by only 12 amino acids [70]. Screening of such libraries can lead to remarkable improvements in activity by using saturation mutagenesis at homologous enzyme positions [71].

As the fitness of a gene will increase more rapidly in a breeding population with high genetic variability that is under the influence of selection, DNA Family Shuffling, an improvement in the breeding technique based on recombination of several homologous sequences, yielded an improvement in activity of about 30 to 80 times higher than when each gene was shuffled independently [72]. Further techniques can create shuffle exons or domains [73], loop regions [74], random truncations [75] or insertions and deletions of codons [76].

Random redesign techniques are being currently used to generate enzymes with improved properties, such as: activity and stability at different pH values and temperatures [77], increased or modified enantioselectivity [78], altered substrate specificity [79], stability in organic solvents [80], novel substrate specificity and activity [81] and increased biological activity of protein pharmaceuticals and biological molecules [82,83]. Directed evolution increased the activity of glyphosate-N-acetyltransferase by 10,000-fold and at the same time, its thermostability increased 5-fold [84]. Proteins from directed evolution work were already on the market in 2000 [85,86]. These were GFP of Clontech and Novo Nordisk’s LipoPrime^®^ lipase.

## 4. Production of Recombinant Proteins in Microbial Hosts

In 2005, there were 4,200 biotechnology companies worldwide. Total revenue amounted to $63 billion. Recombinant DNA technology has been remarkably advanced by their development of efficient and scale-up expression systems to produce enzymes from industrially-unknown microorganisms and other living organisms using industrial organisms such as *E. coli*, *Bacillus subtilis* and other species of *Bacillus*, *Ralstonia eutropha*, *Pseudomonas fluorescens*, *Saccharomyce* *cerevisiae*, *Pichia pastoris*, *Hansenula polymorpha*, and species of *Aspergillus and Trichoderma* [87]. Approximately 90% of industrial enzymes are recombinant versions.

*E. coli* has been extensively used as a recombinant host for many reasons including (i) ease of quickly and precisely modifying the genome; (ii) rapid growth to high cell densities; (iii) ease of culture in cheap media; and (iv) ease of reduction of protease activity. *E. coli* can accumulate heterologous proteins up to 50% of its dry cell weight [88]. However, it has several drawbacks, such as: inability for post-translational modifications, presence of toxic cell wall pyrogens or, sometimes, formation of inclusion bodies containing inactive, aggregated and insoluble heterologous protein. Despite this, high level production and excretion has been obtained with the following heterologous proteins: alkaline phosphatase (PhoA) at 5.2 g/L into the periplasm, and levan fructotransferase LFT at 4 g/L into the medium. In 1993, recombinant processes in *E. coli* were responsible for almost $5 billion worth of products (87). *R. eutropha* produced organophosphohydrolase at 10 g/L [89]. The *P. fluorescens* culture of the Pfenex company is capable of producing 20 g/L of protein [90].

*S. cerevisiae* offers certain advantages over bacteria as a cloning host [91]. This yeast can be grown rapidly in simple media and to a high cell density, can secrete heterologous proteins into the extracellular broth, and its genetics are more advanced than any other eukaryote. Glucose oxidase from *Aspergillus niger* has been produced at 9 g/L by this yeast (87)*.* Despite these advantages, *S. cerevisiae* is sometimes regarded as a less than optimal host for large-scale production of mammalian proteins because of drawbacks such as hyperglycosylation, presence of α-1, 3-linked mannose residues that could cause an antigenic response in patients, and absence of strong and tightly-regulated promoters.

*P. pastoris* has become one of the most extensively used expression systems [87,92]. Over 700 proteins have been produced by this yeast [93]. Production of recombinant proteins reached 22 g/L for intracellular proteins [94] and 14.8 g/L for secreted proteins [95]. Claims have been made that *P. pastoris* can produce up to 30 g/L of recombinant proteins [96]. Among the advantages of this methylotrophic yeast over *S. cerevisiae* are (i) an efficient and tightly-regulated methanol promoter (AOX1) which yields alcohol oxidase at 30% of soluble protein; (ii) less extensive glycosylation, due to shorter chain lengths of N-linked high-mannose oligosaccharides, usually up to 20 residues lacking the terminal α-1,3-mannose linkages [97]; (iii) integration of multicopies of foreign DNA into chromosomal DNA yielding stable transformants; (iv) ability to secrete high levels of foreign proteins; and (v) high-density growth and straight-forward scale-up [98].

Heterologous gene expression in the methylotropic yeast *H. polymorpha* is similar to that of *P. pastoris*. The promoter of the methanol oxidase gene is used to express foreign genes. As with AOX1 in *P. pastoris*, the MOX gene in *H. polymorpha* is highly expressed and tightly-regulated, giving enzyme levels up to 37% of total cell protein [99]. One major difference is that expression of the MOX gene is significantly derepressed in the absence of glucose or during glucose limitation [100] and therefore tight regulation of the MOX promoter is lost in the high glucose conditions usually used for high-biomass fermentations. *H. polymorpha* can produce 1.4 g/L of secreted glucoamylase and 13.5 g/L of phytase.

The development of molecular techniques for production of recombinant heterologous proteins in filamentous fungi has been laborious and contrasts markedly with the success achieved in yeasts. The ability to introduce or delete genes remains difficult, although some advances in transformation have been reported, e.g., restriction enzyme-mediated integration [101] and *Agrobacterium tumefaciens*-Ti plasmid-mediated transformation [102]. Levels of production of non-fungal proteins have been low compared to those of homologous proteins. Different strategies have been developed to overcome these problems, including the construction of protease-deficient strains [103], introduction of a large number of gene copies [104], use of strong fungal promoters, efficient secretion signals [104,105], and gene fusions with a gene which encodes part of or an entire well-secreted protein [105]. Little research has been carried out on glycosylation in molds although hyper-glycosylation does not seem to occur and low-mannose side chains are formed. *Aspergillus awamori* produces 4.6 g/L of glucoamylase from *A. niger*. *Aspergillus oryzae* yields 3.3 g/L of *Mucor* rennin. *Acremonium chrysogenum* produces 4 g/L of *Fusarium* alkaline protease. *Trichoderma reesei* produces 35 g/L of recombinantproteins.A recent entry into the field is *Chrysosporium lucknowense*, which produces high levels of proteins (50–80 g/L), and from which low-viscosity and low-protease mutants have been obtained [106,107].

## 5. Enzymatic Biocatalysis

Biocatalysts have been widely applied in the brewing and food industries for a long time but ar finding new applications in many fields including industrial chemistry [108]. Biocatalysis involves the application of whole microbial cells, cell extracts, purified enzymes, immobilized cells, or immobilized enzymes as catalysts for any of the above mentioned processes [109,110]. As mentioned in the previous sections, its rapid development was due to the recent advances in large-scale genome sequencing, directed evolution, protein expression, metabolic engineering, high throughput screening, and structural biology [111,112].

### 5.1. Technical Applications

Enzymes are very important for processes of industrial, pharmaceutical and biotechnological significance [113]. The total market for industrial enzymes reached $3.3 billion in 2010 and it is estimated to reach a value of 4.4 billion by 2015 [114]. Of these, technical enzymes are typically used as bulk enzymes in the detergent, textile, pulp and paper industries, and in the biofuels industry, among others. Usage for leather and bioethanol is responsible for the highest sales figures. Technical enzymes had revenues of nearly $1.2 billion in 2011 which is expected to reach $1.5 billion in 2015 and $1.7 billion in 2016. The highest sales are expected to be in the biofuels (bioethanol) market [115]. Use of enzymes for foods and beverages is expected to reach $1.3 billion by 2015.

The use of enzymes as detergent additives represents a major application of industrial enzymes. Proteases, lipases, amylases, oxidases, peroxidases and cellulases are added to detergents where they catalyze the breakdown of chemical bonds on the addition of water. To be suitable, they must be active under thermophilic (60 °C) and alkalophilic (pH 9–11) conditions, as well as in the presence of the various components of washing powders.

Proteases constitute over 60% of the global market for enzymes. They are used to produce pharmaceuticals, foods, detergents, leather, silk and agrochemical products. In laundry detergents, they account for approximately 25% of the total worldwide sales of enzymes. The first detergent containing a bacterial protease (“Biotex”) was introduced by Novo Industry A/S (now Novozymes) in 1956. It contained an alcalase produced by *Bacillus licheniformis*. In 1994, Novo Nordisk introduced Lipolase^TM^, the first commercial recombinant lipase for use in a detergent, by cloning the *Humicola lanuginose* lipase into the *A. oryzae* genome. In 1995, Genencor International introduced two bacterial lipases, one from *Pseudomonas mendocina* (Lumafast^TM^), and another from *Pseudomonas alcaligenes* (Lipomax^TM^). An enzyme added recently to detergents is Mannaway^TM^, a *Bacillus* mannanase which removes food stains containing guar gum [116].

In the textile industry, enzymes are also increasingly being used to develop cleaner processes and reduce the use of raw materials and production of waste. The application of cellulases for denim finishing and laccases for decolorization of textile effluents and textile bleaching are the most recent commercial advances [117]. An alternative enzymatic process in the manufacturing of cotton has been recently developed based on a pectate lyase [118]. The process is performed at much lower temperatures and uses less water than the classical method.

Another application of enzymes with increasing importance is the use of lipases, xylanases and laccases in removing pitch (hydrophobic components of wood, mainly triglycerides and waxes) in the pulp industry [119]. A lipase from *Candida rugosa* is used by Nippon Paper Industries to remove up to 90% of these compounds [120]. The use of enzymes as alternatives to chemicals in leather processing has proved successful in improving leather quality and in reducing environmental pollution. Alkaline lipases from *Bacillus* strains, which grow under highly alkaline conditions, in combination with other alkaline or neutral proteases, are currently being used in this industry. Laccases oxidize phenolic and non-phenolic lignin-related compounds as well as environmental pollutants [121]. They are used to detoxify industrial effluents from the paper and pulp, textile, and petrochemical industries, as a medical diagnostic tool, for bioremediation of herbicides, pesticides, and explosives in soil, as a cleaning agent for water purification systems, as a catalyst in drug manufacture and as cosmetic ingredients.

Although cellulases have been widely used in textile applications for many years, these enzymes are gaining additional consideration in the enzyme market owing to their ability in the degradation of lignocellulosic feedstocks. The cost of cellulases is a key issue in achieving a low price conversion of lignocellulosic biomass into biofuels and other products [122,123,124]. Filamentous fungi can produce native cellulases at levels greater than 100 g/L [87]. Enzymes needed to hydrolyze cellulose include (1) endoglucanases, which break down cellulose chains in a random manner; (2) cellobiohydrolases, which liberate glucose dimers from both ends of cellulose chains; and (3) beta-glucosidases, which produce glucose from oligomer chains. *Hypocrea jecorina (Trichoderma reesei)* is the main industrial source of cellulases and hemicellulases used to depolymerize plant biomass to simple sugars [125,126,127]. The overall action of *T. reesei* on cellulosic biomass is limited by its low content of beta-glucosidase. The result is accumulation of cellobiose which limits further breakdown. The expression of the beta-glucosidase gene from *Pericona* sp in *T. reesei* resulted in an increased level of beta-glucosidase, thus increasing overall cellulase activity and action on biomass residues [128]. Cellulases are formed adaptively, and several positive (XYR1, ACE2, HAP2/3/5) and negative (ACE1, CRE1) components involved in this regulation are now known [127]. In addition, its complete genome sequence has been published [129], thus making the organism susceptible to targeted improvement by metabolic engineering. It has recently been reported that the extreme thermophilic bacterium *Caldicelluloseruptor bescil* produces a cellulase/hemicellulase system twice as active as that from *T. reesei* [130].

### 5.2. Enzymes in the Feed Industry

The global market for feed enzymes is a promising segment in the enzyme industry and is expected to reach about $730 million in 2015 [131]. Feed enzymes can increase the digestibility of nutrients, leading to greater efficiency in feed utilization. Also, they can degrade unacceptable components in feed, which are otherwise harmful or of little or no value [132]. Currently, feed enzymes commercially available are phytases, proteases, α-galactosidases, glucanases, xylanases, α-amylases, and polygalacturonases, mainly used for swine and poultry [133]. Developments of heat-stable enzymes, improved specific activity, some new non-starch polysaccharide-degrading enzymes, and rapid, economical and reliable assays for measuring enzyme activity have always been the focus and have been intensified recently. The use of enzymes as feed additives is restricted in many countries by local regulatory authorities [134] and applications may therefore vary from country to country.

### 5.3. Enzymes in Food Processing

Food and beverage enzymes constitute the largest segment of industrial enzymes with revenues of nearly $1.2 billion in 2011 which is expected to grow to $1.8 billion by 2016, at a composed annual growth rate of 10.4% [115].

Lipases are commonly used in the production of a variety of products, ranging from fruit juices, baked foods, and vegetable fermentations to dairy enrichment. Fats, oils and related compounds are the main targets of lipases in food technology. Accurate control of lipase concentration, pH, temperature and emulsion content is required to maximize the production of flavor and fragrance. The lipase mediation of carbohydrate esters of fatty acids offers a potential market for use as emulsifiers in foods, pharmaceuticals and cosmetics. There are three recombinant fungal lipases currently used in the food industry, one from *Rhizomucor miehi*, one from *Thermomyces lanuginosus* and another from *Fusarium oxysporum*; all being produced in *A. oryzae* [135,136].

The major application of proteases in the dairy industry is for the manufacture of cheese. Calf rennin had been preferred in cheese making due to its high specificity, but microbial proteases produced by GRAS microorganisms like *Mucor miehei*, *Bacillus subtilis*, *Mucor pusillus Lindt* and *Endothia parasitica* are gradually replacing it. The primary function of these enzymes in cheese making is to hydrolyze the specific peptide bond (Phe105-Met106) that generates para-k-casein and macropeptides [137]. Production of calf rennin (chymosin) in recombinant *A. niger var awamori* amounted to about 1 g/L after nitrosoguanidine mutagenesis and selection for 2-deoxyglucose resistance [138]. Further improvement was done by parasexual recombination resulting in a strain producing 1.5 g/L from parents producing 1.2 g/L [139]. Four recombinant proteases have been approved by FDA for cheese production [140,141].

Bacterial glucose isomerase, fungal α-amylase and glucoamylase are currently used to produce “high fructose corn syrup” from starch in a $1 billion business. Other enzymes useful in the food industry include invertase from *Kluyveromyces fragilis*, *Saccharomyces* *carlsbergensis* and *S. cerevisiae* for candy and jam manufacture, β-galactosidase (lactase) from *Kluyveromyces lactis*, *K. fragilis* or *Candida pseudotropicalis* for hydrolysis of lactose from milk or whey, and galactosidase from *S. carlsbergensis* for crystallization of beet sugar. “Fructose syrup”, produced by xxylose isomerase on glucose is at a production level of 15 million tons per year [142].

In terms of government regulation, enzymes used in food can be divided into food additives and processing aids. Most food enzymes are considered as processing aids, with only a few used as additives, such as lysozyme and invertase [143]. The processing aids are used during the manufacturing process of foodstuffs, and do not have a technological function in the final food. All these materials are expected to be safe, under the guidance of good manufacturing practice (GMP). The key issue in evaluating safety of enzyme preparations is the safety assessment of the production strain. Only about nine recombinant microorganisms are considered “Generally Recognized As Safe” (GRAS) based on FDA regulations. These are from a relatively small number of bacterial and fungal species primarily *A. oryzae*, *A. niger*, *B. subtilis* and *B. licheniformis*. Olempska-Beer [144] has reviewed the microbial strains engineered for food enzyme production from a security point of view.

### 5.4. Enzymes in Chemical and Pharmaceutical Processes

Successful application of enzymatic processes in the chemical industry depends mainly on cost competitiveness with the existing and well-established chemical methods [145]. Lower energy demand, increased product titer, increased catalyst efficiency, less catalyst waste and byproducts, as well as lower volumes of wastewater streams, are the main advantages that biotechnological processes have as compared to well-established chemical processes. There are estimated to be only around 150 biocatalytic processes currently applied in industry [146]. However, new scientific developments in genomics, as well as in protein engineering, facilitate the tailoring of enzyme properties to increase that number significantly [147,148].

An enzymatic conversion was devised to produce the amino acid L-tyrosine [149]. Phenol, pyruvate, pyridoxal phosphate and ammonium chloride are converted to L-tyrosine using a thermostable and chemostable tyrosine phenol lyase obtained from *Symbiobacterium toebii*. The titer produced was 130 g/L after 30 h with continuous feeding of substrate.

Industrial scale biocatalysis is focused primarily on hydrolases, a few ketoreductases (KREDs), cofactor regeneration and protein stability in organic solvents. The numerous biocatalytic routes scaled up for pharmaceutical manufacturing have been recently reviewed by Bornscheuer *et al*. [112], showing the competitiveness of enzymes versus traditional chemical processes.

Enzymes are useful for preparing beta-lactam antibiotics such as semi-synthetic penicillins and cephalosporins [150]. Beta-lactams constitute 60%–65% of the total antibiotic market.

Preparation of chiral medicines, *i.e.*, the synthesis of complex chiral pharmaceutical intermediates efficiently and economically, is one of the most important applications in biocatalysis. Esterases, lipases, proteases and KREDs (ketoreductases) are widely applied in the preparation of chiral alcohols, carboxylic acids, amines or epoxides, among others [116,151,152].

The inherent inefficiency of kinetic resolution (maximum 50% yield) can be overcome by novel asymmetric reactions catalyzed by improved microbial enzymes which can provide a 100% yield [153]. Asymmetric reduction of tetrahydrothiophene-3-one with a wild-type reductase gave the desired alcohol ((R)-tetrahydrothiophene-3-ol), a key component in sulopenem, a potent antibacterial developed by Pfizer, but only in 80%–90% ee (enantiomeric excess). A combination of random mutagenesis, gene shuffling and ProSAR analysis was used to improve the enantio-selectivity of a ketoreductase towards tetrahydrothiophene-3-one. The best variant increased enantio-selectivity from 63% ee to 99% ee [154].

Atorvastatin, the active ingredient of Lipitor, a cholesterol-lowering drug that had global sales of US$12 billion in 2010, can be produced enzymatically. The process is based on three enzymatic activities: a ketone reductase, a glucose dehydrogenase and a halohydryn dehalogenase. Several iterative rounds of DNA shuffling for these three enzymes, with screening in the presence of iteratively higher concentrations of product, led to a 14-fold reduction in reaction time, a 7-fold increase in substrate loading, a 25-fold reduction in enzyme use, and a 50% improvement in isolated yield [155].

Kinetic resolution of racemic amines is a common method used in the synthesis of chiral amines. Acylation of a primary amine moiety by a lipase is used by BASF for the resolution of chiral primary amines in a multi-thousand ton scale [156]. Recently, asymmetric synthesis from the corresponding chiral ketones, using transaminases, is gaining attention [152]. Some (R)-selective transaminases have been recently discovered using *in silico* strategies for a sequence-based prediction of substrate specificity and enantio-preference [157]. Optically pure (S)-amines were obtained using a recombinant ω-transaminase with 99% ee and 97% yield [158]. These enantiopure amines may find use as inhibitors of monoamine oxidase in the treatment of diverse neurological disorders such as Parkinson’s and Alzheimer’s diseases.

An effective enzymatic process using enzyme evolution was developed by the biotechnology company Codexis, in cooperation with Pfizer, to produce 2-methyl pentanol, an important intermediate for manufacture of pharmaceuticals and liquid crystals [159]. Quite recently, protein engineering expanded the substrate range of transaminases to ketones. In an impressive piece of work developed by Merck and Codexis, the chemical manufacture of sitagliptin, the active ingredient in Januvia which is a leading drug for type 2 diabetes, was replaced by a new biocatalytic process. Several rounds of directed evolution were applied to create an engineered amine transaminase with a 40,000-fold increase in activity [160]. Such a process not only reduced total waste (by 19%), but also increased overall yield (by 13%) and productivity (by 53%). Codexis scientists also developed enzymatic processes for the production of montelukast (Singulair) and silopenem [161]. They also developed an improved LovD enzyme (an acyltransferase) for improved conversion of the cholesterol-lowering agent, lovastatin, to simvastatin [162].

Optically active carboxylic acids have been usually synthesized through different enzymatic routes catalyzed by lipases, nitrilases or hydroxynitrile lyases. Recent advances have improved the efficiency of such procedures. The synthesis of 2-arylpropanoic acids (e.g., ketoprofen, ibuprofen and naproxen) is mainly achieved through the kinetic resolution of racemic substrates by lipases from *Candida antarctica* or *Pseudomonas* sp. A process using a novel substrate, (R,S)-N-profenylazoles, instead of their correspondent esters, proved to be more efficient.

(R)-*o*-chloromandelic acid is a key intermediate for manufacture of Clopidogrel, a platelet aggregation inhibitor, with global sales of $10 billion per year. The asymmetric reduction of methyl o-chlorobenzoylformate with a versatile recombinant carbonyl reductase from *S. cerevisiae* expressed in *E. coli* yielded (R)-*o*-chloromandelate at a concentration of 178 g/L and 99% ee [163].

Application of enzymes in several industrial bioconversions has been broadened by the use of organic solvents replacing water [45,50], an important development in enzyme engineering. Many chemicals and polymers are insoluble in water and its presence leads to undesirable by-products and degradation of common organic reagents. Although switching from water to an organic solvent as the reaction medium might suggest that the enzyme would be denatured, many crystalline or lyophilized enzymes are actually stable and retain their activities in such anhydrous environments. Yeast lipases have been used to catalyze butanolysis in anhydrous solvents to obtain enantiopure 2-chloro- and 2-bromo-propionic acids that are used for the synthesis of herbicides and pharmaceuticals [151]. Another lipase is used in a stereoselective step, carried out in acetonitrile, for the acetylation of a symmetrical diol during the synthesis of an antifungal agent [164].

From a biotechnological perspective, there are many advantages of employing enzymes in organic, as opposed to aqueous, media [165], including higher substrate solubility, reversal of hydrolytic reactions, and modified enzyme specificity, which result in new enzyme activities. On the other hand, enzymes usually show lower catalytic activities in organic than in aqueous solution.

## 6. Conclusion and Future Perspectives

Microorganisms provide an impressive amount of catalysts with a wide range of applications across several industries such as household care, food, animal feed, technical industries, fine chemicals and pharma. The unique properties of enzymes such as high specificity, fast action and biodegradability allow enzyme-assisted processes in industry to run under milder reaction conditions, with improved yields and reduce waste generation.

However, naturally occurring enzymes are often not suitable for such biocatalytic processes without further tailoring or redesign of the enzyme itself in order to fine-tune substrate specificity activity or other key catalytic properties.

Recent advances in genomics, metagenomics, proteomics, efficient expression systems and emerging recombinant DNA techniques have facilitated the discovery of new microbial enzymes from nature (through genome and metagenome) or by creating (or evolving) enzymes with improved catalytic properties.

The ongoing progress and interest in enzymes provide further success in areas of industrial biocatalysis. The next years should see a lot of exciting developments in the area of biotransformations. There are many factors influencing the growing interest in biocatalysis which include enzyme promiscuity, robust computational methods combined with directed evolution and screening technologies to improve enzyme properties to meet process prospects, the application of one-pot multistep reactions using multifunctional catalysts and the *de novo* design and selection of catalytic proteins catalyzing any desired chemical reaction.

Many future investigations will use combinations of engineered and *de novo* designed enzymes coupled with chemistry to generate more (and most likely new) chemicals and materials from cheaper (and renewable) resources, which will consequently contribute to establishing a bio-based economy and achieving low carbon green growth.
